# Editorial: Gut microbiota modulation to mitigate stress-induced functional changes

**DOI:** 10.3389/frmbi.2025.1731851

**Published:** 2026-01-20

**Authors:** Ruoting Yang, Rasha Hammamieh

**Affiliations:** Walter Reed Army Institute of Research, Silver Spring, MD, United States

**Keywords:** gut-brain axis, microbiome, modulation of microbiota, stress pathophysiology, stress therapeutic

## The gut-brain axis and stress

The gut-brain axis is a complex, bidirectional signaling network involving the central, autonomic, and enteric nervous systems, alongside endocrine, immune, and metabolic mediators. Its equilibrium is essential for health. Stress, defined as a state of threatened homeostasis, activates the hypothalamic-pituitary-adrenal (HPA) axis, leading to sustained elevations of glucocorticoids (e.g., cortisol) during chronic exposure. Environmental perturbations in endocrine signaling can also lead to microbial changes; for example, altered photoperiod reduces melatonin and reshapes the cecal microbiota and breast muscle inflammation in broilers [Yu et al.].

The gastrointestinal (GI) tract is a primary target of these stress hormones. Preclinical and clinical studies show that these hormones can significantly alter the composition and function of the gut microbiota, linking stress-induced physiological changes directly to conditions such as anxiety, depression, and irritable bowel syndrome (IBS). In this Research Topic, this theme is evident across models of exertional heat stroke [Xuan et al.], traumatic brain injury [Rusling et al.], and chronic stress-induced obesity with cognitive decline [Liu et al.].

## Stress-induced dysbiosis and its consequences

HPA axis activation releases signaling molecules (e.g., CRF and glucocorticoids) that directly alter intestinal motility, secretion, and visceral sensitivity. These alterations modulate the growth conditions for commensal microbes, favoring certain taxa and leading to dysbiosis.

Perhaps the most critical consequence—and one of the most studied—is the impairment of intestinal barrier function. Reduced expression of tight junction proteins leads to increased intestinal permeability, or “leaky gut”. This allows microbial components, notably lipopolysaccharides (LPS), to translocate from the gut lumen in the bloodstream. This triggers a pro-inflammatory cascade. Because these circulating cytokines can cross the blood-brain barrier, they are directly implicated in promoting neuroinflammation, which in turn contributes to mood and cognitive disturbances.

## The mechanistic roles of the microbiota

Gut microbes influence the brain and behavior through several interconnected pathways; however, it is worth asking which of these are truly significant. Much attention has been given to the production of neuroactive metabolites. For instance, certain Lactobacillus species can produce GABA, and some Clostridium species can stimulate serotonin (5-HT) synthesis. However, it is still a matter of intense debate whether this microbial GABA or 5-HT significantly impacts central neurotransmitter pools, rather than just acting locally in the gut.

A more robust pathway appears to be through short-chain fatty acids (SCFAs), especially butyrate, which is produced by the microbial fermentation of dietary fiber. Butyrate is fascinating because it functions as a histone deacetylase (HDAC) inhibitor. This is not just a biochemical curiosity; it means that butyrate can epigenetically modulate gene transcription in colonocytes and immune cells, thereby directly reducing inflammation. Butyrate also appears crucial for the integrity of both the intestinal and blood-brain barriers. Interest in SCFA-related pathways is also reflected in clinical meta-analyses of putative SCFA-producing taxa in spinal cord injury [Zhong et al.].

Additionally, the vagus nerve acts as a direct, non-hormonal communication highway. Microbial metabolites can stimulate its afferent pathways, sending signals straight to the brainstem. This pathway is compelling, but it is notoriously difficult to study in humans, leaving many questions about its relative importance unanswered.

## Microbiota-targeted interventions

This flurry of mechanistic data has, understandably, led to a boom in “psychobiotic” interventions. Probiotics (live microorganisms) are the most direct example, and their reputation has often outpaced the empirical data. Nevertheless, specific strains of Lactobacillus (e.g., L. helveticus) and Bifidobacterium (e.g., B. longum) have shown promise in reducing psychological distress in some clinical trials. In line with this, probiotic supplementation has prevented stress-impaired spatial learning in rats and amplified the effects of environmental enrichment [Flynn et al.]. Beyond classical psychobiotics, several studies in this Research Topic evaluate nutritional and feed-based approaches that reshape microbial communities and host physiology in animal models [Jiang et al., Li et al., Yang et al.].

Prebiotics (e.g., FOS/GOS) offer a more subtle approach by feeding the beneficial bacteria we already have to enhance endogenous SCFA production. Of course, all these points go back to diet. Fiber-rich and fermented food diets support a diverse ecosystem, whereas the typical Western diet, which is processed and high in fat, appears to be almost designed to promote dysbiosis. Diet composition can modify stress responses itself; for example, omega-6 enrichment altered microbiome dynamics and physiological outcomes in rodent models of acute traumatic psychological stress and blast traumatic brain injury (TBI) [3].

## Challenges and future directions

Despite these promising results, the field is struggling with significant hurdles. The high inter-individual variability in microbiota composition means that a “one-size-fits-all” probiotic is unlikely to be effective for everyone. Furthermore, trial outcomes are wildly inconsistent, and the mechanisms by which these probiotics work (or fail to work) are poorly understood. What the field desperately needs, in our view, is a move beyond simple correlational studies. We need longitudinal, multi-omics studies to establish causal links. Simply put, progress will depend on developing personalized approaches that tailor interventions to an individual’s unique microbial and physiological profile. Contributions to this Research Topic also point toward stratification and controllability. For example, baseline gut microbiota features predicted chemotherapy-induced neutropenia duration in leukemia patients [Huang et al.], while conceptual and experimental work argues for host-controllable healthy microbiomes and tractable, multi-strain model systems for testing mechanisms [Bouchez et al., Maloney et al.].

## Conclusion

In conclusion, the gut microbiota is unequivocally a key regulator of the host stress response. Its disruption is a direct contributor to the physiological and psychological sequelae of chronic stress. Modulating the microbiota is therefore a compelling therapeutic strategy. While significant challenges in personalization remain, continued investigation into these mechanisms holds immense potential for novel therapeutics for stress-related disorders.

